# Decoding the Genetic Basis of Mast Cell Hypersensitivity and Infection Risk in Hypermobile Ehlers-Danlos Syndrome

**DOI:** 10.3390/cimb46100689

**Published:** 2024-10-17

**Authors:** Purusha Shirvani, Arash Shirvani, Michael F. Holick

**Affiliations:** Section of Endocrinology, Diabetes, Nutrition and Weight Management, Department of Medicine, Boston University School of Medicine, Boston, MA 02118, USA

**Keywords:** Ehlers-Danlos syndrome, mast cell activation syndrome, mast cell hypersensitivity, infection, whole-genome sequencing

## Abstract

Hypermobile Ehlers-Danlos syndrome (hEDS) is a connective tissue disorder marked by joint hypermobility, skin hyperextensibility, and tissue fragility. Recent studies have linked hEDS with mast cell activation syndrome (MCAS), suggesting a genetic interplay affecting immune regulation and infection susceptibility. This study aims to decode the genetic basis of mast cell hypersensitivity and increased infection risk in hEDS by identifying specific genetic variants associated with these conditions. We conducted whole-genome sequencing (WGS) on 18 hEDS participants and 7 first-degree relatives as controls, focusing on identifying genetic variants associated with mast cell dysregulation. Participants underwent clinical assessments to document hEDS symptoms and mast cell hypersensitivity, with particular attention to past infections and antihistamine response. Our analysis identified specific genetic variants in MT-CYB, HTT, MUC3A, HLA-B and HLA-DRB1, which are implicated in hEDS and MCAS. Protein–protein interaction (PPI) network analysis revealed significant interactions among identified variants, highlighting their involvement in pathways related to antigen processing, mucosal protection, and collagen synthesis. Notably, 61.1% of the hEDS cohort reported recurrent infections compared to 28.5% in controls, and 72.2% had documented mast cell hypersensitivity versus 14.2% in controls. These findings provide a plausible explanation for the complex interplay between connective tissue abnormalities and immune dysregulation in hEDS. The identified genetic variants offer insights into potential therapeutic targets for modulating mast cell activity and improving patient outcomes. Future research should validate these findings in larger cohorts and explore the functional implications of these variants to develop effective treatment strategies for hEDS and related mast cell disorders.

## 1. Introduction

Ehlers-Danlos syndrome (EDS) comprises a group of inherited connective tissue disorders characterized by joint hypermobility, skin hyperextensibility, and tissue fragility [1,2]. Among the various subtypes, hypermobile EDS (hEDS) is the most common form, yet its genetic basis remains elusive [3,4]. Recent studies have highlighted a significant association between hEDS and mast cell activation syndrome (MCAS), suggesting a complex interplay between connective tissue disorders and immune dysregulation [5,6]. Mast cell hypersensitivity has emerged as a common phenotype in patients with hEDS. A study by Seneviratne et al. found that 66% of patients with hEDS also met the diagnostic criteria for MCAS [7]. Similarly, studies reported a high prevalence of MCAS in hEDS patients, with overlapping symptoms including chronic pain, fatigue, and gastrointestinal disturbances [8,9]. These findings indicate that mast cell dysfunction may play a crucial role in the multisystemic manifestations of hEDS.

Recent studies have provided additional evidence supporting the hypothesis of a connection between hEDS and MCAS. A cohort analysis conducted in an allergy/immunology clinic found that a significant number of patients with suspected MCAS also had diagnoses of hEDS or hypermobility spectrum disorder (HSD), alongside primary immune deficiencies such as immunoglobulin deficiency [10]. Of the 974 patients analyzed, 10% had both MCAS and hEDS/HSD, and 19% had a combination of MCAS, hEDS/HSD, and immunoglobulin deficiency [10]. This suggests a notable overlap between these conditions and highlights the need to explore underlying contributors to chronic inflammation and tissue injury in hEDS patients [10]. The potential connection between hEDS and mast cell-related disorders may be explained by the presence of mast cell mediators, especially tryptase and histamine, which can affect multiple organ systems [10,11]. Immunohistochemistry analysis has identified an increased chymase (a mast cell product) content in undamaged skin of patients with signs of connective tissue disorders [7,12]. This suggests an intrinsic alteration in mast cell function or distribution in hEDS patients. Interestingly, patients with hEDS have also been observed to have an increased susceptibility to a wide range of infections. Boileau et al. [13] reported a higher incidence of recurrent infections, particularly urinary tract infections, in hEDS patients compared to the general population. Moreover, a functional analysis of fibroblasts from patients with joint hypermobility syndrome (JHS) and hypermobile Ehlers-Danlos syndrome (hEDS) revealed that these patients exhibited altered immune responses, including decreased cytokine production, which may contribute to their increased risk of infections [14].

The relationship between mast cell hypersensitivity and infection susceptibility is complex and bidirectional. Mast cells play a crucial role in innate immunity and host defense against pathogens [15,16,17]. However, dysregulated mast cell activation can lead to excessive inflammation and tissue damage, potentially increasing vulnerability to infections [15,16,17,18]. Conversely, recurrent infections may trigger chronic mast cell activation, creating a vicious cycle of immune dysregulation [19,20,21].

Recent epidemiological data further support the association between hEDS and MCAS. A study using the National Inpatient Sample found that nearly one in three patients diagnosed with MCAS have a comorbid diagnosis of hEDS [21]. This association appeared to increase over time with the increasing diagnosis of MCAS, suggesting a growing recognition of the overlap between these conditions [7,21].

Despite these observations, the genetic underpinnings of hEDS, mast cell hypersensitivity, and infection susceptibility in these patients remain poorly understood. While several genes have been implicated in other EDS subtypes, the causative factors and mechanisms for hEDS and its associated comorbidities are largely unknown [3,4]. This knowledge gap highlights the need for comprehensive genetic studies to elucidate the molecular basis of these interrelated phenomena [1,2].

To explore these observations further, we conducted whole-genome sequencing in 18 participants with hypermobile Ehlers-Danlos syndrome (hEDS) alongside their 7 first-degree relatives as controls. Our study aims to explore the hypothesis that mast cell hypersensitivity or activation is more prevalent in hEDS participants compared to the general population, and that they also experience higher infection rates. Additionally, we seek to uncover genetic variations associated with hEDS by identifying alterations related to mast cell function and immune system regulation. This research has the potential to illuminate the complex pathophysiology of hEDS and its associated immune dysregulation, providing insights into the genetic basis of these interconnected conditions.

## 2. Materials and Methods

### 2.1. Study Participants and Ethical Considerations

We initiated a clinical research program approved by the Boston University Medical Center’s Institutional Review Board (IRB) to address the needs of participants with hypermobile Ehlers-Danlos syndrome (hEDS). This program provides a dedicated clinic and research initiative for the diagnosis, treatment, and study of hEDS. Participants and their relatives were invited to participate through phone calls, mail, and email. An information letter was sent to the primary contact (potential index case) and any accompanying family members, outlining the study details and providing an opportunity for questions prior to their visit.

Upon arrival at the General Clinical Research Unit (GCRU), participants were provided with a consent form to review and sign in the presence of a team member. This consent allowed for a physical examination, documentation of physical findings with photographs, and the collection of blood and saliva samples from adult participants, children, and relatives. Once consented, participants received a copy of the consent form for their records, and biological samples were collected for DNA extraction.

### 2.2. Clinical Assessment

During their visit, participants completed a comprehensive questionnaire detailing medical history and symptoms related to hypermobile Ehlers-Danlos syndrome (hEDS). They were asked about signs and symptoms following the 2017 International Classification for hEDS criteria for themselves and their affected children, with responses recorded and retained. Physical examinations were conducted to evaluate physical signs of hEDS, including joint hypermobility using the Beighton score, skin characteristics, and other features such as unexplained striae, piezogenic papules, atrophic scarring, dental crowding, arachnodactyly, and mitral valve prolapse [1,2,3,22].

To assess mast cell hypersensitivity or dysregulation, participants underwent a detailed clinical evaluation focusing on symptoms commonly associated with mast cell activation. These symptoms included dermatological manifestations such as flushing, hives, itching, and swelling; gastrointestinal issues like abdominal pain, cramping, diarrhea, and nausea; respiratory symptoms including wheezing, shortness of breath, and nasal congestion; cardiovascular signs such as palpitations, dizziness, and episodes of low blood pressure; and neurological symptoms like headaches, fatigue, and cognitive difficulties [5,6,7,8,9,16,17]. Participants’ past medical history was evaluated, with particular attention to recurrent infections and any hospital admissions, as well as their response to antihistamine therapy, to identify patterns indicative of mast cell dysregulation.

For children, especially those under the age of 5, the assessment of hypermobile Ehlers-Danlos syndrome (hEDS) presents unique challenges. The 2017 diagnostic criteria for hEDS, primarily developed for adults, require adaptation when applied to younger populations [23,24,25,26]. The Pediatric Working Group of the International Consortium on Ehlers-Danlos Syndromes has developed a new diagnostic framework to address these challenges, recognizing that joint hypermobility is highly prevalent in young children and often decreases with age [23]. For children aged 5 and older, the Beighton score is used to assess generalized joint hypermobility, with a score of 6 or more indicating significant hypermobility [2,23,24,25,26]. However, for children under 5, clinical evaluation focuses on developmental milestones, family history, and the presence of symptoms such as musculoskeletal pain, skin extensibility, and other connective tissue abnormalities [2,17,25].

The framework allows for a fluid diagnosis, acknowledging that some children may initially present with generalized joint hypermobility and later meet the full criteria for hEDS as they mature. This approach ensures that children receive appropriate monitoring and management, even if they do not yet meet the full diagnostic criteria for hEDS [23,24,25,26,27]. By integrating these diagnostic criteria and assessment methods, we aimed to accurately identify both hEDS and mast cell hypersensitivity in our cohort, differentiating them from other conditions with similar presentations. This process is crucial for understanding the role of mast cell dysregulation in hEDS and guiding future research and treatment strategies.

### 2.3. DNA Extraction and Whole-Genome Sequencing

Genomic DNA was extracted from collected blood and saliva samples. Whole-genome sequencing (WGS) was performed at the Molecular Biology Core at the Dana–Farber Cancer Institute as previously described [28]. Following initial quality control, an automated PCR-free library preparation was conducted using the Swift 2S protocol (Swift Biosciences, Ann Arbor, MI, USA). Sequencing was performed on an Illumina HiSeq 2000 platform to achieve 60X coverage with 100 bp paired-end reads.

### 2.4. Data Processing and Quality Control

The quality of sequencing reads was assessed using FastQC (Version 0.12.0). The BWA-MEM algorithm was employed to align reads to the GRCh38 human reference genome. Processed data were uploaded to Illumina BaseSpace for comprehensive analysis. Variant calling was performed using Illumina’s Isaac-based WGS v4 app in BaseSpace Sequence Hub, generating VCF files for small variants. These VCF files were subsequently imported into the Variant Interpreter for basic annotation and filtering of PASS variants [28].

### 2.5. Data Analysis

Data analysis, including alignment to the GRCh38 reference genome, variant calling, and annotation, was performed using validated software v2.17 from Illumina (Illumina, Inc., San Diego, CA, USA), including the DRAGEN platform [28]. This comprehensive analysis pipeline ensured thorough and accurate processing of the genomic data.

### 2.6. Gene Selection and Variant Filtering

In our study, variant filtering was concentrated on small genetic variants, utilizing the Variant Interpreter (Strelka 2.9.2, Illumina, Inc., San Diego, CA, USA) to identify pathogenic, likely pathogenic, and variants of uncertain significance. We implemented an innovative strategy to capture genetic variations relevant to hypermobile Ehlers-Danlos syndrome (hEDS) and mast cell sensitivity or activation. Initially, we filtered genetic variations that were exclusively present at least in the 15% of our hEDS subjects (*n* = 3) and absent in the control group. This curated list of hEDS-specific variations was then used to pinpoint genetic alterations potentially associated with hEDS or specifically related to mast cell activity in hEDS patients.

To achieve this, we compiled two lists, one comprising known genes involved in mast cell activation syndrome (MCAS) and another including genes associated with collagen metabolism in other EDS types that share phenotypic traits with hEDS. The list of genes potentially involved in mast cell dysregulation and hypersensitivity was derived from a thorough review of relevant literature and gene panels available on the Illumina BaseSpace platform, as summarized in Table 1.

Our approach involved a multi-step analysis using a protein–protein interaction (PPI) network to explore the relationship between the discovered genetic variations unique to our hEDS subjects and the known genes related to MCAS and other EDS types.

Step 1: We utilized the PPI network to identify potential pathways within the list of hEDS-specific genes where variations were found only in our hEDS subjects and not in controls.

Step 2: The PPI network was expanded to include known genes from other EDS types that share some phenotypes with hEDS, aiming to discern any relationships between these genes and those identified in our study.

Step 3: Finally, we focused on capturing MCAS-specific genetic variants within the hEDS cohort by examining their potential interactions with established MCAS-related genes through the PPI network.

This comprehensive methodology aimed not only to identify specific genetic variations associated with hEDS but also to uncover potential genetic links between hEDS and mast cell dysregulation, thereby contributing valuable insights into the genetic basis of these interconnected conditions.

## 3. Results

In this study, we conducted a genomic evaluation of 18 participants diagnosed with hypermobile Ehlers-Danlos syndrome (hEDS) and 7 first-degree relatives who served as controls. The mean age of the hEDS participants was 17.4 years, with ages ranging from 1 to 60 years, and 38.8% (*n* = 7) of the participants were female. In the control group, the mean age was 55.8 years, with ages ranging from 39 to 73 years, and 42.8% (*n* = 3) were female. A significant portion of the hEDS cohort, 61.1% (*n* = 11), reported a history of recurrent infections, in contrast to 28.5% (*n* = 2) in the control group. Additionally, 72.2% (*n* = 13) of the hEDS participants had a documented medical history of mast cell hypersensitivity, compared to only 14.2% (*n* = 1) among the controls.

### 3.1. Sequencing Quality and Coverage

The whole-genome sequencing (WGS) achieved an average genome coverage depth ranging from 48X to 55X. The total number of reads varied between 850 million and 1 billion base pairs. The alignment quality was robust, with 84% to 86% of reads/bases aligning well to the reference genome. Furthermore, the sequencing data demonstrated high quality, with 91% to 94% of bases achieving a Q30 score, indicating a low error rate and reliable sequencing results.

### 3.2. Genetic Variants and Filtering Approaches

Each subject had between 5 and 6.2 million total genetic variants. Variant filtering focused on small variants, utilizing the Variant Interpreter to identify pathogenic, likely pathogenic, and variants of uncertain significance. All variants passed quality controls, excluding any classified as benign or likely benign. Initially, we filtered genetic variations that were exclusively present at least in the 15% of our hEDS subjects (*n* = 3) and absent in the control group.

### 3.3. Pathways Potentially Involved in Hypermobile Ehlers-Danlos Syndrome (hEDS)

**Figure 1:**
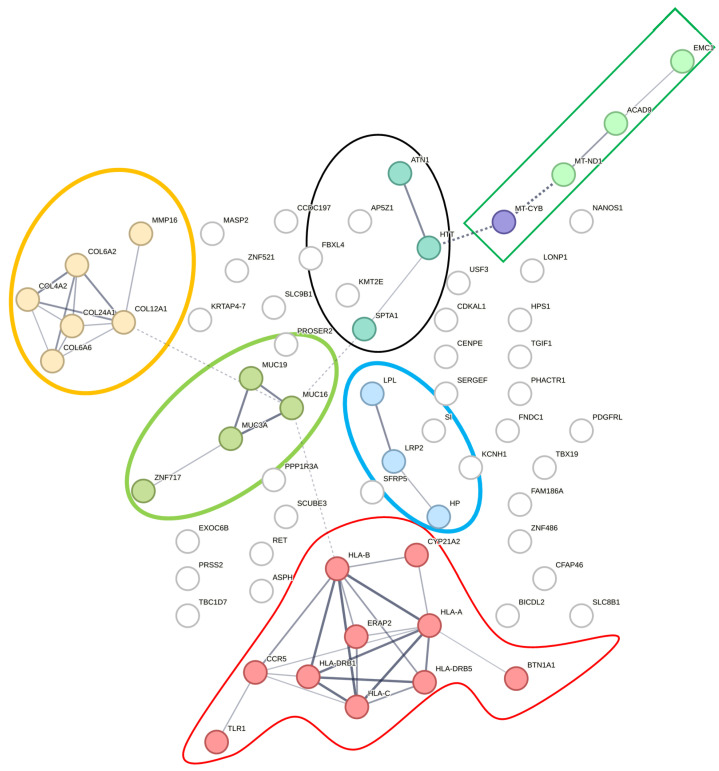
Protein–protein interaction network highlighting mast cell hypersensitivity pathways. This figure illustrates the protein–protein interaction network, which demonstrated significantly more interactions than expected for a random set of proteins, with a PPI enrichment *p*-value of 1.49 × 10^−10^. The Markov cluster algorithm (MCL) identified at least six distinct clusters within the network. MCL is a method used to cluster proteins based on their interaction patterns within a protein-protein interaction network. This approach helps identify groups of proteins that interact more frequently with each other than with those outside the group, suggesting functional relatedness. The first cluster is involved in antigen processing and the presentation of endogenous peptide antigens and MHC protein complexes (red border). The second cluster relates to the defective GALNT3 causing hyperphosphatemic familial tumoral calcinosis (HFTC), including genes such as MUC3A, MUC16, MUC19, and ZNF717 (green circle). These MUC genes are major glycoprotein components of mucus gels, providing a protective barrier against particles and infectious agents at mucosal surfaces and potentially involved in ligand binding and intracellular signaling. The third cluster is associated with collagen chain trimerization and extracellular matrix structural constituents conferring tensile strength, including genes such as COL4A2, COL6A2 and MMP16 (yellow circle). The fourth cluster relates to retinoid and cholesterol metabolism, including genes such as LPL and LRP2 (blue circle). The fifth cluster is associated with mitochondrial complex I assembly model OXPHOS system, including genes such as MT-ND1 and ACAD9 (green rectangle). The last cluster relates to triplet repeat expansion, including genes such as SPTA1 (black circle). This refers to proteins encoded by a gene which has a triplet repeat expansion, i.e., the increase of triplet (trinucleotide) repeats within the gene sequence. The length of such repeats is frequently polymorphic, and there is often a correlation between repeat length and disease severity.

We utilized the PPI network to identify potential pathways within the list of hEDS-specific genes where variations were found only in our hEDS subjects and not in controls (Figure 1). This approach focused on variants present in at least 15% (*n* = 3) of the hEDS participants but absent in the control group. The network demonstrated significantly more interactions than expected for a random set of proteins, with a PPI enrichment *p*-value of 1.49 × 10^−10^. The Markov cluster algorithm (MCL) identified at least six distinct clusters within the network. MCL is a method used to cluster proteins based on their interaction patterns within a protein–protein interaction network. This approach helps identify groups of proteins that interact more frequently with each other than with those outside the group, suggesting functional relatedness.

The first cluster was involved in antigen processing and presentation of endogenous peptide antigens and MHC protein complexes, including genes such as HLA-A, HLA-B, HLA-C, HLA-DRB1, and HLA-DRB5. The second cluster was related to defective GALNT3 causing HFTC, including genes such as MUC3A, MUC16, MUC19, and ZNF717. These MUC genes are major glycoprotein components of mucus gels, providing a protective barrier against particles and infectious agents at mucosal surfaces and potentially involved in ligand binding and intracellular signaling. The third cluster was associated with collagen chain trimerization and extracellular matrix structural constituents conferring tensile strength. The fourth cluster related to retinoid and cholesterol metabolism, including genes such as LPL, LRP2, and HP. The fifth cluster was associated with the mitochondrial complex I assembly model OXPHOS system, including genes such as MT-CYB, MT-ND1, EMC1, and ACAD9. The last cluster related to triplet repeat expansion, including genes such as SPTA1, HTT, and ATN1. This refers to proteins encoded by a gene which has a triplet repeat expansion, i.e., the increase of triplet repeats within the gene sequence. The length of such repeats is frequently polymorphic, and there is often a correlation between repeat length and disease severity. This network analysis supports the hypothesis of a higher rate of mast cell hypersensitivity and increased susceptibility to infection in hEDS participants, highlighting potential genetic contributors to these conditions (Figure 1).

### 3.4. Identifying Relevant Genetic Variations in Hypermobile Ehlers-Danlos Syndrome (hEDS)

**Figure 2:**
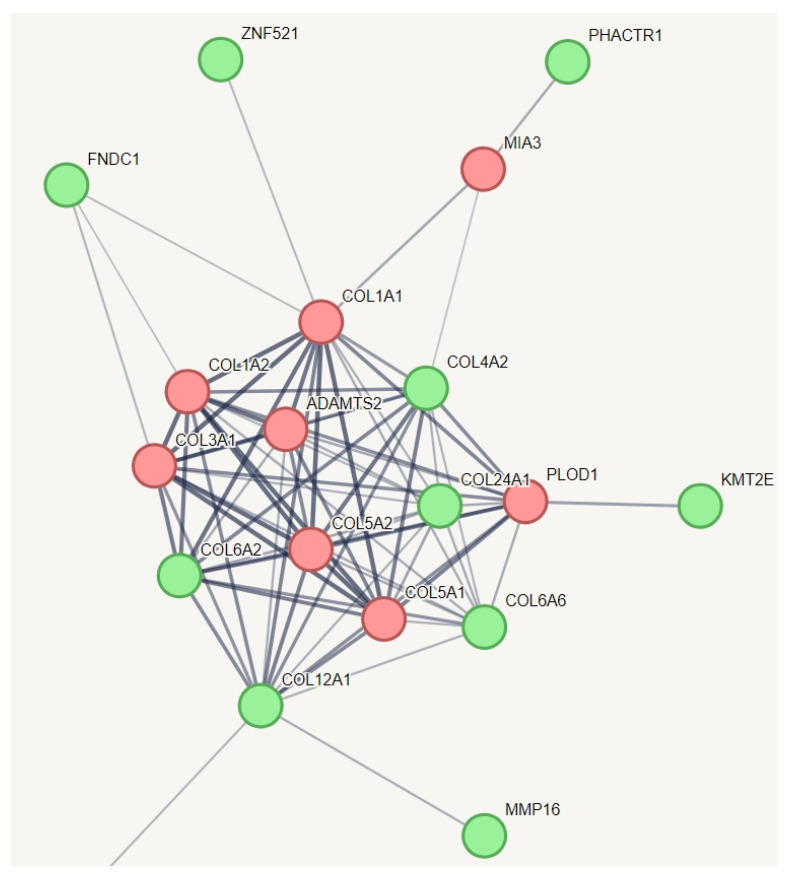
Protein–protein interaction network capturing hEDS-specific genes with potential relationships to established EDS genes. This figure illustrates the protein–protein interaction network, which showed significantly more interactions than expected for a random set of proteins, with a PPI enrichment *p*-value of 1.49 × 10^−10^. The red nodes represent the known genes associated with different types of EDS, while the green nodes represent genes with variations specific to hEDS subjects that have relationships with these known genes. All of these genes, except PHACTR1, are involved in collagen chain trimerization and extracellular matrix structural constituents conferring tensile strength. PHACTR1 (phosphatase and actin regulator 1) binds actin monomers (G actin) and plays a role in various processes, including the regulation of actin cytoskeleton dynamics, actin stress fibers formation, cell motility and survival, tubule formation by endothelial cells, and regulation of PPP1CA activity. It is also involved in the regulation of cortical neuron migration and dendrite arborization. To simplify the figure, pathways related to HLA and information repeated from Figure 1 have been removed from this pathway.

We employed an innovative strategy to capture the relevant genetic variation for hEDS. In a protein–protein interaction (PPI) network of all genes with specific variations unique to hEDS, we incorporated known genes from other types of Ehlers-Danlos syndrome (EDS) that share some phenotypes with hEDS (Figure 2). This creative approach aimed to identify which of these genes might have a relationship with the established EDS genes. The results indicated that all known genes across various types of EDS are interconnected, as expected, and they collectively function within a network related to collagen metabolism. Notably, we identified additional genes within this network, including COL6A2, COL6A6, COL4A2, COL12A1, COL24A1, KMT2E, FNDC1, ZNF521, PHACTR1, and MMP16. Among them, zinc finger protein 521 is a transcription factor that can act as either an activator or a repressor depending on the context. It is involved in BMP signaling and regulates the immature compartment of the hematopoietic system. It associates with SMADs in response to BMP2, leading to the activation of BMP target genes. As a transcriptional repressor, it interacts with EBF1, a transcription factor involved in B-cell lineage specification, preventing EBF1 from binding DNA and activating target genes. Zinc finger protein 521 belongs to the Krueppel C2H2-type zinc finger protein family. All of these genes, except PHACTR1, are involved in collagen chain trimerization and extracellular matrix structural constituents conferring tensile strength. PHACTR1 (phosphatase and actin regulator 1) binds actin monomers (G actin) and plays a role in various processes, including the regulation of actin cytoskeleton dynamics, actin stress fibers formation, cell motility and survival, tubule formation by endothelial cells, and regulation of PPP1CA activity. It is also involved in the regulation of cortical neuron migration and dendrite arborization.

### 3.5. Identifying Relevant Genetic Variations in Association with MCAS in hEDS

In a protein–protein interaction (PPI) network of all genes with specific variations unique to hEDS, we incorporated known genes related to MCAS (Figure 2). This creative approach aimed to identify which of these genes might have a relationship with the established MCAS genes. The red and green nodes represent known genes associated with mast cell activation syndrome (MCAS), while the yellow nodes represent genes with variations specific to hEDS subjects that have relationships with these known MCAS genes. The results indicate that all known genes for MCAS are interconnected, as expected. The red nodes are involved in pathways related to hematopoietic or lymphoid organ development, whereas the yellow nodes participate in inflammatory responses and the positive regulation of interleukin-10 production. The results demonstrate that all known genes related to mast cell activation syndrome or mast cell hypersensitivity are interconnected, as anticipated. We identified additional genes within this network, including ADGRE2, TLR1, CCR5, HP, ERAP2, SFRP5, GATA4, RUNX2, KMT2E, RET, TPSAB1, TBX19, and ZNF521. Notably, ZNF521, a transcription factor, was also identified in the pathway depicted in Figure 2. It plays a role alongside RUNX2 in regulating osteoblast differentiation.

### 3.6. Key Genes and Variations Associated with hEDS

**Figure 3:**
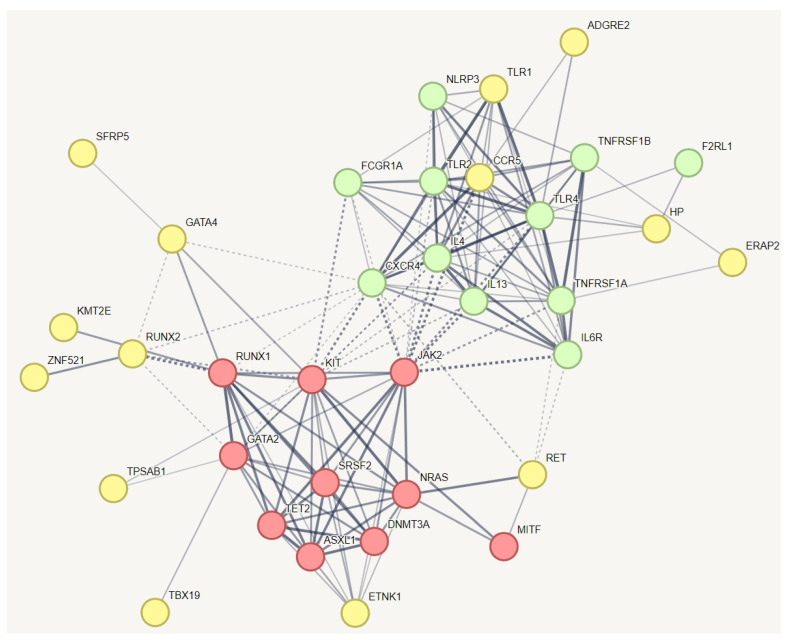
Protein–protein interaction network capturing MCAS-specific genetic variants in hEDS with potential relationships to established MCAS genes. This figure illustrates the protein–protein interaction network, which demonstrated significantly more interactions than would be expected for a random set of proteins, with a PPI enrichment *p*-value of 1.0 × 10^−16^. The red and green nodes represent known genes associated with mast cell activation syndrome (MCAS), while the yellow nodes represent genes with variations specific to hEDS subjects that have relationships with these known MCAS genes. The red nodes are involved in pathways related to hematopoietic or lymphoid organ development, whereas the yellow nodes participate in inflammatory responses and the positive regulation of interleukin-10 production. The results demonstrate that all known genes related to mast cell activation syndrome or mast cell hypersensitivity are interconnected, as anticipated. We identified additional genes within this network, including TLR1, RET, HP, ZNF521, and CCR5. Notably, ZNF521, a transcription factor, was also identified in the pathway depicted in Figure 2. It plays a role alongside RUNX2 in regulating osteoblast differentiation. To simplify the figure, pathways related to HLA and information repeated from Figure 1 and Figure 2 have been removed from this pathway.

To identify genetic variations relevant to hypermobile Ehlers-Danlos syndrome (hEDS) and mast cell sensitivity or activation, we employed a multi-step strategy. Initially, we filtered for genetic variations present in at least 15% of our hEDS subjects (*n* = 3) but absent in the control group. We then compiled a list of genes associated with these variations and explored their interactions with known hEDS-related genes (Figure 2) and those involved in mast cell activation syndrome (MACS) (Figure 3). The results are summarized in Table 2, which presents seven gene clusters identified in this study. Each gene cluster potentially plays a role in hEDS or mast cell dysregulation associated with hEDS. The final column of Table 2 indicates the number of hEDS subjects carrying variations in the genes within each cluster. Notably, 16 hEDS subjects (88%) had variations in genes linked to mast cell activation syndrome. From all studied clusters, we identified five key genes—MT-CYB, HTT, MUC3A, HLA-B, and HLA-DRB1—with 94% of hEDS subjects carrying at least one variation from these genes. Table 3 summarizes these variations, highlighting the p.(Leu119Trp) variant in the HLA-B gene at chr6:31356429 as the most common among hEDS participants. This novel missense variant involves a nucleotide change from GAG (reference) to CCA (alternate), potentially altering the HLA-B protein structure, which is crucial for antigen presentation and immune response modulation. The discovery of this variant in over half of the hEDS participants, especially those with mast cell hypersensitivity, and its absence in controls, suggests its potential as a biomarker for hEDS. The structural changes induced by this variant may affect the protein’s ability to present antigens, thereby influencing immune recognition and response.

## 4. Discussion

Our study offers significant insights into the genetic basis of hypermobile Ehlers-Danlos syndrome (hEDS) and its association with mast cell hypersensitivity and increased susceptibility to infections. By conducting whole-genome sequencing (WGS) on a cohort of hEDS participants and their relatives, we identified several genetic variations that may contribute to these complex clinical manifestations.

The identification of specific genetic variants associated with mast cell dysregulation in hEDS, such as those in the MT-CYB, HTT, MUC3A, HLA-B, and HLA-DRB1genes, underscores the potential role of these variants in the pathophysiology of hEDS. These findings align with previous studies that have highlighted the prevalence of mast cell activation syndrome (MCAS) in hEDS, suggesting a shared genetic basis for these conditions. Notably, our research has identified a novel variant, p.(Leu119Trp), in the HLA-B gene. This missense variant, not previously reported, could potentially alter the structure of the HLA-B protein, a crucial component in antigen presentation and immune response modulation. The discovery of this variant in more than half of the hEDS participants, particularly those with mast cell hypersensitivity, and its absence in the control group suggests its potential as a biomarker for hEDS. The structural changes induced by this variant might affect the protein’s ability to present antigens, thereby influencing immune recognition and response. This finding highlights the role of the immune system in the pathophysiology of hEDS and opens avenues for targeted therapeutic interventions. The presence of HLA-B variants, in particular, may indicate an immune-mediated component to the disorder, as HLA-B is involved in antigen presentation and immune response modulation [47]. Research has shown that HLA-B variants can influence the immune system’s ability to recognize and respond to pathogens [53,54], potentially leading to altered immune responses in hEDS. This aligns with studies reporting increased susceptibility to infections in hEDS, possibly due to compromised immune function [13,14]. The presence of MUC genes, known for their role in forming protective mucus barriers [55], highlights a potential mechanism for the increased susceptibility to infections observed in hEDS participants. This is consistent with studies that have reported altered immune responses and increased infection rates in hEDS populations [13,14]. MT-CYB is involved in mitochondrial function and energy production, which are crucial for the high-energy demands of bone formation and maintenance. Mitochondria play a role in mast cell activation, where adequate ATP levels are essential for FcεRI-dependent activation [56]. This supports our finding that variations in MT-CYB may contribute to mast cell dysregulation observed in hEDS patients. HTT has been shown to participate in cytokine production processes in mast cells, affecting inflammatory responses [57]. This suggests that HTT variants may contribute to immune dysregulation seen in hEDS.

Additionally, our research identified variants in genes such as ZNF521, ZNF717, RUNX2, and MMP16, which are implicated in bone mineralization and osteoblast differentiation, potentially explaining the higher fracture rates observed in hEDS patients. ZNF521 is a transcription factor known to repress osteoblastic differentiation [58]. Studies have shown that ZNF521 inhibits the maturation of osteoblasts by reducing the expression of key osteogenic markers like collagen I, alkaline phosphatase, and osteopontin, leading to decreased matrix mineralization [58]. This repression could contribute to weaker bone structure in hEDS patients, increasing fracture risk even at an early age. RUNX2 is a critical transcription factor for bone development, regulating genes involved in osteoblast differentiation and bone matrix production [59]. RUNX2 promotes the maturation of chondrocytes and their transdifferentiation into osteoblasts, essential for normal bone formation during development [59]. Variants affecting RUNX2 function can disrupt these processes, potentially leading to compromised bone integrity and increased fracture susceptibility in hEDS individuals. MMP16 plays a role in remodeling the extracellular matrix by degrading collagen and other matrix components [60]. Alterations in MMP16 activity can affect the structural integrity of connective tissues, contributing to the fragility observed in hEDS. This gene’s involvement in collagen turnover highlights its potential impact on maintaining bone strength.

Mitochondrial dysfunction can impair osteoblast activity, leading to compromised bone strength and increased fracture risk. A study by Guo et al. [61] highlighted the importance of mitochondrial DNA variants in influencing bone mineral density (BMD) and the risk of osteoporosis, suggesting that mitochondrial dysfunction could impact bone health. HLA-B and HLA-DRB1 are part of the major histocompatibility complex involved in immune response regulation. Variants in these genes have been linked to osteoporosis and reduced bone mineral density, suggesting a role in bone health through immune-mediated pathways [62]. The presence of HLA-B variants implies an immune-mediated component to hEDS, given their role in antigen presentation and immune response modulation.

The identification of these genetic variants aligns with previous studies highlighting the prevalence of MCAS in hEDS, suggesting a shared genetic basis for these conditions. Mast cells are known to influence bone metabolism by regulating osteoclastogenesis and osteoblast activity [63]. Dysregulated mast cell activation can lead to excessive inflammation and tissue damage, potentially increasing vulnerability to fractures [63].

Overall, these genetic variants provide a plausible explanation for the complex interplay between connective tissue abnormalities and immune dysregulation in hEDS. The identification of these variants not only enhances our understanding of the genetic basis of hEDS but also opens avenues for targeted therapeutic interventions aimed at modulating mast cell activity and improving patient outcomes. Future studies should focus on validating these findings in larger cohorts and exploring the functional implications of these variants to develop effective treatment strategies for hEDS and associated mast cell disorders.

Our approach to variant filtering, focusing on genes involved in mast cell hypersensitivity and excluding variants present in controls, allowed us to pinpoint genetic factors that may be unique to hEDS. This is supported by the clustering of variants in pathways related to connective tissue integrity and immune function, providing a plausible link between genetic predisposition and clinical phenotype.

Despite the promising findings, our study has several limitations that need to be acknowledged. The relatively small sample size is a significant limitation, as it may affect the generalizability of our results. Although our study design included first-degree relatives as controls to account for shared genetic and environmental factors, the limited number of participants restricts the statistical power and robustness of our conclusions. Future research should aim to include larger, more diverse cohorts to validate these findings and explore the genetic landscape of hypermobile Ehlers-Danlos syndrome (hEDS) more comprehensively. Additionally, while we have identified genetic associations that suggest a potential role in mast cell dysregulation and infection susceptibility in hEDS, functional studies are necessary to elucidate the precise biological mechanisms involved. The complexity of hEDS suggests that other genetic or environmental factors may also contribute to its pathophysiology. Therefore, integrating genomic data with environmental and lifestyle factors could provide a more holistic understanding of hEDS.

Another limitation is the age disparity between hEDS participants and control groups, which may introduce confounding variables related to age-dependent immune function differences. Future studies should strive to include age-matched controls to minimize these potential biases. Finally, while our use of whole-genome sequencing (WGS) provides comprehensive coverage of genetic variants, it is essential to complement these findings with biochemical analyses such as cytokine profiling or mast cell activation markers in blood or saliva. These additional data could offer insights into systemic inflammation and immune dysregulation in hEDS. In conclusion, while our study lays the groundwork for understanding the genetic basis of hEDS, further research is crucial for confirming these associations and developing targeted therapeutic strategies.

## 5. Conclusions

Our study provides insights into the potential genetic basis of hypermobile Ehlers-Danlos syndrome (hEDS) and its associated comorbidities, such as mast cell hypersensitivity and increased infection susceptibility. By identifying certain genetic variants and pathways through whole-genome sequencing, we have established a foundation for future research that could inform the development of targeted therapies and improved diagnostic criteria for hEDS and related mast cell disorders. However, while our findings are promising, they are not definitively conclusive.

The identification of specific genetic variants, including those in MT-CYB, HTT, MUC3A, HLA-B, and HLA-DRB1, suggests a possible role of these genes in the pathophysiology of hEDS. These findings align with previous studies that indicate a prevalence of mast cell activation syndrome (MCAS) in hEDS and suggest a potential shared genetic basis for these conditions. Notably, the presence of HLA-B variants implies an immune-mediated component to the disorder, given their role in antigen presentation and immune response modulation.

Nevertheless, our study has limitations that must be acknowledged. The relatively small sample size means that these findings should be viewed as preliminary. Further research with larger cohorts is necessary to validate these results and to explore the functional implications of these variants more thoroughly. Additionally, while we have identified potential genetic associations, the complexity of hEDS suggests that other genetic or environmental factors may also play significant roles. Continued investigation is crucial for developing effective diagnostic and treatment strategies and enhancing our understanding of hEDS.

## 6. Patents

This research has led to the development of innovative findings that have been protected under U.S. intellectual property law. Specifically, the work reported in this manuscript has resulted in a U.S. Provisional Patent Application, filed under the application number 63/701,801. This patent application covers the novel genetic variants identified in association with hypermobile Ehlers-Danlos syndrome (hEDS) and their potential use as biomarkers or therapeutic targets. The protection of these discoveries underscores their significance and potential impact on advancing the understanding and treatment of hEDS and related conditions.

## Figures and Tables

**Table 1:** The genes involved in mast cell sensitivity. This table summarizes the genes associated with mast cell hypersensitivity, mast cell activation, and related disorders, along with their functions and references.

Gene	Associated Conditions/Functions
**KIT (CD117)**	Encodes the receptor for stem cell factor (SCF), crucial for mast cell development and function and mastocytosis, pathological mast cell activation [29]
JAK2	Mast cell activation, V617F mutation associated with pathological activation [29,30]
MRGPRX2	Mas-related G-protein coupled receptor, involved in non-IgE-mediated mast cell activation [31]
ADGRE2	Mast cell activation, influence on mast cell reactivity [32]
PLCG2	Signal transduction, involved in cold-induced urticaria [32]
TET2	Systemic mastocytosis, candidate tumor suppressor gene [29]
NRAS	Aggressive systemic mastocytosis [29]
IL13 AND IL4	Allergic diseases, polymorphism associated with increased risk of systemic mastocytosis [33]
TLR1	Innate immunity, implicated in mast cell activation [34]
FCER1	The high-affinity IgE receptor, plays a central role in allergic reactions and mast cell activation and high-affinity IgE receptor [35,36]
H1R, H2R, H3R, H4R	Histamine receptors, mediate various physiological responses including allergic reactions [36]
TNF RECEPTORS (TNFR1 AND TNFR2)	Receptors for tumor necrosis factor, involved in inflammatory signaling [30,33,36]
GATA2	Hematopoiesis and immune regulation, transcription factor [37]
HRH4	Histamine signaling, inhibition of full-length receptor function in mast cells [29]
NLRP3	Inflammatory responses, associated with IL-1β production [38]
IDH1I AND DH2	Cancer metabolism, involved in immune responses [39]
DNMT3A	Epigenetic regulation, associated with hematological malignancies [40]
TLR2 AND TLR4	Toll-like receptors involved in recognizing bacterial components and mediating immune responses and their polymorphisms associated with systemic mastocytosis [33]
IL-4R AND IL6R	Receptor for interleukin-4, involved in promoting Th2 immune responses. Allergic diseases, gain-of-function polymorphism associated with mastocytosis [33]. Receptor for interleukin-6, involved in inflammatory responses and polymorphisms studied in mastocytosis [33]
RASGRP4	Systemic mastocytosis, involved in mast cell activation [29,41]
ASXL1	Chromatin modification, associated with hematological malignancies [42,43]
**CYP26B1**	Mediates inhibition of mast cell activation by fibroblasts to maintain skin-barrier homeostasis [44]
ETNK1	Phospholipid metabolism, involved in immune responses [45]
ZNF521	Zinc finger transcription factor, involved in immune responses
SRSF2	RNA splicing, associated with hematological malignancies [43]
PDGFRA	Pathological mast cell activation, associated with systemic mastocytosis [29]
CBL	Cytokine-independent mast cell activation, associated with systemic mastocytosis [29]
MITF	Transcription factor involved in mast cell development [36]
CXCR4	Chemokine receptor involved in cell migration and immune responses [36]
PAR2	Protease-activated receptor, involved in inflammation and pain signaling [36]
FCGR1A	Fc gamma receptors are widely expressed on a variety of immune cells and play a myriad of regulatory roles in the immune system [46]
HLA-B	This gene is part of the major histocompatibility complex (MHC) and plays a critical role in immune response. Variations in HLA-B have been associated with various immune-mediated conditions [47], which could influence mast cell activity through immune modulation.
RUNX1	Variations in RUNX1 have been associated aggressive systemic mastocytosis [43,48]
TPSAB1	Hereditary alpha-tryptasemia, mast cell activation syndrome [49]
CD177	This is a specific neutrophil activation marker [50,51,52]

**Table 2:** Key genes in different cluster were involved in hypermobile Ehlers-Danlos syndrome (hEDS) and mast cell activation syndrome (MCAS). The table highlights the potential involvement of these gene clusters in hEDS or mast cell dysregulation. Additionally, the table includes a column indicating the number of hEDS subjects carrying variations in each cluster, providing insights into the prevalence of these genetic variations among the study participants.

Clusters	Genes	hEDS Subjects with Variations % (*n*)
Antigen processing and presentation of endogenous peptide antigens and MHC protein complex	CYP21A2, CCR5, ERAP2, BTN1A1, TLR1, HLA-A, HLA-B, HLA-C, HLA-DRB1, and HLA-DRB5.	88% (16)
Defective GALNT3 causes HFTC	MUC3A, MUC16, MUC19, and ZNF717.	72% (13)
Retinoid and cholesterol metabolism	LPL, LRP2, and HP.	27% (5)
Mitochondrial complex I assembly model OXPHOS system	MT-CYB, MT-ND1, EMC1, and ACAD9.	55% (10)
Triplet repeat expansion	SPTA1, HTT, and ATN1.	55% (10)
Collagen chain trimerization	COL6A2, COL6A6, COL4A2, COL12A1, COL24A1, KMT2E, COL24A1, FNDC1, ZNF521, PHACTR1, and MMP16.	77% (14)
Mast cell activation syndrome	ADGRE2, TLR1, CCR5, HP, ERAP2, SFRP5, GATA4, RUNX2, KMT2E, RET, TPSAB1, TBX19, and ZNF521.	88% (16)
Selected genes with more variation among the hEDS	MT-CYB, HTT, MUC3A, HLA-B, and HLA-DRB1	94% (17)

**Table 3:** The specific genetic variations identified within the key genes associated with hypermobile Ehlers-Danlos syndrome (hEDS) and mast cell activation syndrome (MCAS). This includes a detailed list of five genes—MT-CYB, HTT, MUC3A, HLA-B and HLA-DRB1—wherein 94% of hEDS subjects carried at least one variation. The table highlights the most common variant, p.(Leu119Trp) in the HLA-B gene, which involves a nucleotide change from GAG to CCA. This variant was notably prevalent among hEDS participants, particularly those with mast cell hypersensitivity, suggesting its potential role as a biomarker for hEDS. The table provides a comprehensive overview of these genetic variations and their distribution among the study cohort.

Variations	VAF ^†^ in HEDS	Genes	Allele Frequency	HGVS/SNP	Consequence	PolyPhen Prediction ^††^
**MT:15607:G**	0.11	MT-CYB	G = 0.08	c.861A>G(p.(Lys287=)) rs193302996	Synonymous	likely pathogenic/benign *
**MT:15452:A**	0.13	MT-CYB	A = 0.17	p.(Leu236Ile) rs193302994	Missense	tolerated— low confidence likely pathogenic/benign
**MT:15043:A**	0.22	MT-CYB	A = 0.06	c.297G>A(p.(Gly99=)) rs193302985	Synonymous	likely pathogenic/benign *
**MT:14905:A**	0.11	MT-CYB	A = 0.07	c.159G>A(p.(Met53=)) rs193302983	Synonymous	likely pathogenic/benign *
**MT:14783:C**	0.16	MT-CYB	C = 0.02	c.37T>C(p.(Leu13=)) rs193302982	Synonymous	likely pathogenic *
**7:100957985:** **100957984:** **CCAGCCAGACCC**	0.22	MUC3A	Unknown	p.(Ser2070_His2071 insThrLysThrProSer)	Inframe insertion	Not Reported
**7:100954094:G**	0.11	MUC3A	G = 0.008	p.(His772Arg) rs933695519	Missense	deleterious— low confidence
**7:100953370:A**	0.08	MUC3A	A = 0.07	p.(Ala531Thr) rs1490623108	Missense	deleterious— low confidence
**6:32589726:G**	0.02	HLA-DRB1	G = 0.04	p.(Leu6Pro) rs201726340	Missense	deleterious
**6:32584379:T**	0.02	HLA-DRB1	T = 0.01	rs775308685	Splice acceptor	Not Reported
**6:32584218:** **32584219:** **CT**	0.02	HLA-DRB1	Unknown	p.(Ala87Glu)	Missense	Deleterious
**6:32584185:** **32584184:TC**	0.02	HLA-DRB1	Unknown	p.(Gln99AspfsTer31)	Frameshift	Not Reported
**6:32584175:** **32584176**	0.02	HLA-DRB1	Unknown	p.(Ala102ArgfsTer25)	Frameshift	Not Reported
**6:32584158:T**	0.13	HLA-DRB1	T = 0.00	p.(Tyr107Ter) rs11554463	Stop gained	Not Reported
**6:32584108:T**	0.02	HLA-DRB1	T = 0.1	rs200689965	Splice donor	
**6:32581834:** **32581836:CTC**	0.02	HLA-DRB1	Unknown	p.(Gln125Glu)	Missense/ Splice region	Tolerated
**6:32581575:C**	0.02	HLA-DRB1	C = 0.02	p.(Pro212Ala) rs1136846	Missense	deleterious
**6:32580276:G**	0.02	HLA-DRB1	G = 0.03	rs28732251	Splice region/ Intron variant	Not Reported
**6:31356429:** **31356431:CCA**	0.25	HLA-B	Unknown	p.(Leu119Trp)	Missense	deleterious— low confidence
**6:31356280:A**	0.08	HLA-B	A = 0.01	p.(Arg169Leu) rs12697943	Missense	deleterious— low confidence
**4:3160307:T**	0.05	HTT	T = 0.002	p.(Thr1260Met) rs34315806	Missense	Deleterious/pathogenic/benign
**4:3074939:** **3074939**	0.05	HTT	delG = 0.00	p.(Gln38HisfsTer63) rs1560535226	Frameshift	Not Reported
**4:3074932:** **3074936**	0.05	HTT	0.004	p.(Gln36ProfsTer45) rs1338001820	Frameshift	Not Reported
**4:3074930:** **3074929:ACA**	0.05	HTT	0.00	p.(Gln38dup) rs1553909026	Inframe insertion	Not Reported
**4:3074883:** **3074882:** **CAGCAGCAGCAG**	0.11	HTT	Unknown	p.(Gln35_Gln38dup)	Inframe insertion	Not Reported
**4:3074880:** **3074879:** **CAGCAGCAG**	0.02	HTT	Unknown	p.(Gln36_Gln38dup)	Inframe insertion	Not Reported

^†^ VAT: variant allele frequency. ^††^ PolyPhen: polymorphism phenotyping. PolyPhen predicts the possible impact of an amino acid substitution on the structure and function of a human protein using straightforward physical and comparative considerations. * The variant is documented as likely pathogenic/benign in the ClinVar database.

## Data Availability

Data will be available beginning 9 months and ending 24 months after article publication upon reasonable request to mfholick@bu.edu.
